# Inferring microbial interaction networks from metagenomic data using SgLV-EKF algorithm

**DOI:** 10.1186/s12864-017-3605-x

**Published:** 2017-03-27

**Authors:** Mustafa Alshawaqfeh, Erchin Serpedin, Ahmad Bani Younes

**Affiliations:** 1 Bioinformatics and Genomic Signal Processing Lab, ECEN Dept., Texas A&M University, College Station, TX, 77843-3128 USA; 2 AE Dept., Khalifa University, Abu Dhabi, UAE

**Keywords:** Microbial interaction network, Extended Kalman filter, Metagenomics, SgLV-EKF algorithm

## Abstract

**Background:**

Inferring the microbial interaction networks (MINs) and modeling their dynamics are critical in understanding the mechanisms of the bacterial ecosystem and designing antibiotic and/or probiotic therapies. Recently, several approaches were proposed to infer MINs using the generalized Lotka-Volterra (gLV) model. Main drawbacks of these models include the fact that these models only consider the measurement noise without taking into consideration the uncertainties in the underlying dynamics. Furthermore, inferring the MIN is characterized by the limited number of observations and nonlinearity in the regulatory mechanisms. Therefore, novel estimation techniques are needed to address these challenges.

**Results:**

This work proposes SgLV-EKF: a stochastic gLV model that adopts the extended Kalman filter (EKF) algorithm to model the MIN dynamics. In particular, SgLV-EKF employs a stochastic modeling of the MIN by adding a noise term to the dynamical model to compensate for modeling uncertainties. This stochastic modeling is more realistic than the conventional gLV model which assumes that the MIN dynamics are perfectly governed by the gLV equations. After specifying the stochastic model structure, we propose the EKF to estimate the MIN. SgLV-EKF was compared with two similarity-based algorithms, one algorithm from the integral-based family and two regression-based algorithms, in terms of the achieved performance on two synthetic data-sets and two real data-sets. The first data-set models the randomness in measurement data, whereas, the second data-set incorporates uncertainties in the underlying dynamics. The real data-sets are provided by a recent study pertaining to an antibiotic-mediated Clostridium difficile infection. The experimental results demonstrate that SgLV-EKF outperforms the alternative methods in terms of robustness to measurement noise, modeling errors, and tracking the dynamics of the MIN.

**Conclusions:**

Performance analysis demonstrates that the proposed SgLV-EKF algorithm represents a powerful and reliable tool to infer MINs and track their dynamics.

## Background

The microbiota, a conglomeration of all the bacteria living on/in the human body, is now being extensively studied in order to understand its relevance to the host. Interestingly, it has been suggested in several works that the maintenance of a stable microbial ecosystem is necessary for a healthy life [1]. For instance, a disruption of the stable state of the microbiome, referred to as ‘dysbiosis’, is directly linked to obesity [2–4], diabetes [5], inflammatory bowel disease (IBD) [6] and cancer [7, 8].

Even though the bacteria have been recognized as playing a key role in defining the health and disease states, their study has represented a challenge in the past due to several reasons. First, the bacteria were mainly studied through cultivation. Many bacterial groups were neither known earlier nor cultivated in a large number in a laboratory setting. Second, in vitro measurements do not match real in vivo values because the laboratory conditions do not match the environment of the host [9]. However, recent advances in high-throughput sequencing have overcome these limitations. At present, the sequencing technologies provide the researchers with cross-sectional and longitudinal microbial compositions in different environments.

In particular, longitudinal microbial studies are important because they offer an insight into the dynamics of the bacterial community and its response to external perturbations [10]. In addition to its importance to understand the variations in bacterial populations, such observational studies are promising to discover the regulation mechanisms which are essential to identify bacterial groups that may cause or protect against diseases [11]. Therefore, time series analysis tools are crucial to exploit the temporal information embedded into the time series data.

Bacterial communities comprise a vast number of species with complex relationships including mutualism, competition, parasitism, commensalism, amenalism and neutralism [12]. These interactions can be mediated by natural competition for space and resources or via some symbiotic relationships. For example, substances secreted by one species may be metabolized by another [13, 14]. Additionally, members of bacterial communities can interact indirectly through the immune system [15]. Identifying these interactions is crucial to understand the ecological communities and the underlying regulation activities between microbes. For example, the depletion of a species may affect other species that depend on it for their survival. As an additional example, the oppositional and symbiotic interactions between species contribute to the development and resistance of pathogens [16].

Various methods have been proposed to infer the microbial interaction network (MIN) [12, 17]. These methods can be broadly divided into similarity-based methods and dynamic-based methods. Similarity-based approaches employ a similarity measure to score the pairwise relationship between each pair of microbes. Two microbes are considered to have an interaction if the pairwise similarity score exceeds a predefined threshold. Popular methodologies for constructing similarity-based networks are the correlation coefficient and local similarity analysis (LSA) [18–22]. While these methods are computationally efficient, they present several drawbacks. Firstly, they identify only pairwise relations. Therefore, complex interactions in microbial communities are not captured. Secondly, similarity-based networks are undirected. This means that the inferred interactions are assumed to be bidirectional with equal strengths. However, this represents an invalid biological assumption. Third, a similarity-based approach treats the time series data as a static snapshot, and hence it ignores the temporal dependencies.

On the other hand, dynamic methods overcome these drawbacks and go beyond identifying only the interaction network to build predictive models that enable tracking the bacterial composition over time and their response to external perturbations [12]. Constructing such a model presents two major phases: (*i*) model selection phase, which aims to determine a set of equations to identify the structure of the system; (*ii*) parameter estimation phase, or commonly referred to as system identification, which determines the unknown parameters of the model from the observed data. A common approach in dynamical modeling is to use ordinary differential equations (ODEs). An example of ODE-based dynamical models that have been employed to characterize the microbial interaction network is the generalized Lotka-Volterra (gLV) model [11, 23–25]. gLV has been extensively used due to the following two main features of gLV equations. Firstly, the model parameters directly capture the growth rates and pairwise interactions between all species in the system. Secondly, the gLV model can be extended to account for external stimuli such as the introduction of probiotics, antibiotics or changes in diet [11]. However, ODE-based models consider only the uncertainty caused by the noise in the measurements. Therefore, the randomness in the dynamical model is not considered by such models.

In general, estimating the unknown parameters is embedded within the optimization framework that aims to minimize the error between the model’s output and the experimental data. The proposed optimization techniques are broadly divided into integral-based methods and regression-based methods. Integral-based methods are iterative algorithms that search the parameter space for an optimal set. At each iteration, the ODEs are solved via numerical integration to compute the difference between the model output and the available data. The primary drawbacks of integral-based algorithms are the computational burden required to solve the ODEs and the convergence failure due to the integration breakdown [26].

To reduce the computational complexity, regression techniques approximate the derivative terms in the ODE model from the observed data, thereby, converting the ODEs into a regular multivariate regression system. For instance, the parameters of a linearized (via logarithmic transformation) version of the gLV model were estimated by the ridge regression in [11] and the sparse linear regression in [24]. The linearization step restricts the bacterial abundance levels to be strictly positive. This assumption is biologically invalid since it is possible that some bacteria may be totally depleted in some samples. Generally, regression-based methods are computationally efficient and scalable for very large dimensional data [27, 28]. However, their performance relies on the accuracy of the estimated derivatives. Therefore, without a proper denoising preprocessing step, the slope approximation may perform poorly due to the overfitting problem [29]. Additionally, for fast varying observations, an intelligent algorithm is required to track the variation in data and provide an accurate estimate of the derivatives. Estimating the model’s parameters is a challenging task due to the following factors: (a) The number of the unknown parameters is much larger than the available observations; (b) The underlying regulation mechanisms that govern the microbial interaction network are nonlinear. The aforementioned literature about inferring the MIN from time series data has not specifically dealt with these two challenges.

To address the challenges mentioned above, we propose a stochastic-based dynamical model that encodes the uncertainties in both the measurements and the dynamics to compensate for modeling errors and capture the complex interactions among the microbiota. Moreover, we propose EKF to jointly estimate the states of the stochastic model and its parameters. EKF is selected because of the following two features. First, EKF can handle the nonlinearities in the dynamic model or the observation model or both via linearization about the current mean and variance. Second, EKF performs the estimation recursively which renders the EKF as a suitable approach for inferring a large number of parameters from a limited number of observations [30]. Although EKF has had success in several biological applications such as gene regulatory networks, signaling pathways and metabolic networks [31–33], it has not been applied to estimate the microbial interaction network from metagenomic time series data. We refer to the combination of the stochastic gLV model with EKF to estimate its parameters as the SgLV-EKF algorithm. The main contributions of this work can be summarized as:

- We improve the conventional modeling of MINs from a nonlinear ODE dynamic model to a more general nonlinear stochastic model to compensate for uncertainties in the model and/or observations.
- We propose the EKF, which has not been proposed in the context of microbial interaction networks, to infer the bacterial interaction network. The EKF is selected due to its inherent ability to estimate the parameters of nonlinear interactions from limited number of observations.
- Comprehensive simulation studies corroborate the fact that the proposed approach outperforms Nelder and Stein’s algorithm in terms of robustness to measurement noise, modeling errors, computational efficiency, and tracking the dynamics of the microbial interaction network.

## Methods

### System model

In this paper, the MIN is modeled as a nonlinear dynamic stochastic system that captures the dynamics of the bacterial abundance level as follows:

1 $$ \begin{array}{rcl} x_{i}(k+1) &=& f_{i}({\boldsymbol{x}}(k))+ w_{i}(k),\\ y_{i}(k) &=& x_{i}(k)+ v_{i}(k), \end{array}  $$

where *i*=1,…,*n* is the state index, *k*=1,…,*M* represents the time-step, *M* is number of measurement time points, ***x***(*k*)∈ℜ^*n*^ denotes the system state vector, and ***y***(*k*)∈ℜ^*n*^ stands for the observation vector. In particular, *y*_*i*_(*k*) and *x*_*i*_(*k*) represent the measured and the actual relative abundance level of the *i* ^*th*^ bacteria at time *k*, respectively. The microbial interaction network containing *n* bacteria is described by the nonlinear function ***f***=[*f*_1_,*f*_2_,…,*f*_*n*_]^*T*^, where *f*_*i*_ is defined in terms of the discrete-time differential equation (4). Variables ${\boldsymbol {w}}(k)\thicksim \mathcal {N}({\boldsymbol {0}},{\boldsymbol {Q}}(k))$ and ${\boldsymbol {v}}(k)\thicksim \mathcal {N}({\boldsymbol {0}},{\boldsymbol {R}}(k))$ represent the zero-mean white Gaussian process noise and measurements noise, respectively, with covariance matrices given by

2 $$ \begin{array}{rcl} E\left\{{\boldsymbol{w}}(k) {\boldsymbol{w}}^{T}(j)\right\} &=& {\boldsymbol{Q}}(k) \delta_{kj},\\ E\left\{{\boldsymbol{v}}(k) {\boldsymbol{v}}^{T}(j)\right\} &=& {\boldsymbol{R}}(k) \delta_{kj},\\ E\left\{{\boldsymbol{w}}(k) {\boldsymbol{v}}^{T}(j)\right\} &=& {\boldsymbol{0}} \end{array}  $$

where *E*{.} denotes the expectation operator and *δ*_*kj*_ denotes the Kronecker delta function:

3 $$ \delta_{kj} = \left\{\begin{array}{ll} 0 & {if }~ k\neq j\\ 1 &{if }~ k=j \end{array}\right..   $$

### Generalized Lotka-Volterra model

The gLV model is a first order nonlinear system of differential equations. In its discrete form, the gLV is represented as a group of first order nonlinear difference equations that relate the dissimilarity between the abundance levels of species at time *t* with respect to time *t*−1.

Let {*x*_*i*_(*t*);*i*=1,…,*n*} be the relative abundance level of the *i* ^*th*^ bacteria at time *t* whose intrinsic growth rate is *g*_*i*_. Moreover, let *c*_*ij*_ represent the strength of the influence of microbe *i* onto bacteria *j* (a.k.a., the ‘interaction coefficient’). The gLV model is defined by means of the following differential equations:

4 $$ \frac{\mathrm{d}}{\mathrm{d}t} x_{i}(t) = g_{i} x_{i}(t) + x_{i}(t) \sum_{j=1}^{n}{c_{ij}x_{j}(t) }.  $$

The above framework was extended to model the effects of external perturbations (e.g., antibiotics, diets) onto the microbial community structure [11]. This was obtained by adding another term to (4) which modulates the influence of each stimulating source into each member of the ecosystem. Mathematically, let *ε*_*il*_ represent the ‘sensitivity’ of the *i* ^*th*^ microbe in response to the *l* ^*th*^ stimuli with signal strength *u*_*l*_. The resulting gLV model is captured by [11]:

5 $$ {\begin{aligned} \frac{\mathrm{d}}{\mathrm{d}t} x_{i}(t) &= g_{i} x_{i}(t) + x_{i}(t) \sum_{j=1}^{n}{c_{ij}x_{j}(t)} + x_{i}(t) \sum_{l=1}^{L}{\epsilon_{il}u_{l}(t)}. \end{aligned}}  $$

We remark in passing that a simplified gLV model was previously employed in [24] to characterize the dynamics of the gut microbiome considering only the interaction between various species. Particularly, the simplistic gLV model is formulated as:

6 $$ \frac{\mathrm{d}}{\mathrm{d}t} x_{i}(t) = x_{i}(t) \sum_{j=1}^{n}{c_{ij}x_{j}(t) },  $$

where the intrinsic growth rate is ignored compared to (4).

### Kalman filter and extended Kalman filter

This section reviews the key features of the Kalman filter and then focuses on formulating the EKF for estimating both the states and parameters of the state space model.

#### Kalman filter

Under certain conditions, e.g., linearity of model and Gaussian noise, the Kalman filter represents an optimal filter of the system state in the presence of measurement errors. Let assume that the dynamics of a discrete-time system is governed by the following linear model:

7 $$ {\boldsymbol{x}}(k+1)= {\boldsymbol\Phi}_{k} {\boldsymbol{x}}(k)+ {\boldsymbol\Gamma}_{k} {\boldsymbol{u}} (k) + {\boldsymbol\Lambda}_{k} {\boldsymbol{w}}(k),  $$

and the observation model is given by

8 $$ {\boldsymbol{y}}(k) = {\boldsymbol{\Psi}}_{k} {\boldsymbol{x}}(k) + {\boldsymbol{v}}(k),  $$

where *k* is a time-step index, ***x***(*k*)∈ℜ^*n*^ represents the system state vector, and ***y***(*k*)∈ℜ^*n*^ stands for the observation vector. The variable *n* denotes the number of states. Variables ${\boldsymbol {w}}(k)\thicksim \mathcal {N}({\boldsymbol 0},{\boldsymbol {Q}}(k))$ and ${\boldsymbol v}(k)\thicksim \mathcal {N}({\boldsymbol 0},{\boldsymbol {R}}(k))$ represent the zero-mean multivariate Gaussian noise in the process and measurements, respectively. The initial state, and the noise vectors at each step are all assumed to be mutually independent.

The discrete-time Kalman filter assumes the following steps:

- *Initialization*: at *k*=0 and for given initial states ${\hat {\boldsymbol {x}}}^{-}(0)={\boldsymbol {x}}_{0}$, the initial value of the covariance matrix is given by:
  9 $$ \begin{aligned} {\boldsymbol{P}}^{-}(0) = & {\boldsymbol{P}}_{x_{0}x_{0}} = \\ &E\left\{\left({\boldsymbol{x}}(0)-{\boldsymbol{x}_{0}}\right)\left({\boldsymbol x}(0)-{\boldsymbol{x}_{0}}\right)^{T}\right\}, \end{aligned}  $$
  where the superscript (−) denotes a-priori value.
- *Gain*: compute the Kalman gain matrix
  10 $${} {\boldsymbol{K}}(k) = {\boldsymbol{P}}^{-}(k) {\boldsymbol{\Psi}}_{k}^{T} \left[{\boldsymbol{\Psi}}_{k} {\boldsymbol{P}}^{-}(k) {\boldsymbol{\Psi}}_{k}^{T} + {\boldsymbol{R}}(k)\right]^{-1}.  $$
- *Update*: update the state estimate ${\hat {\boldsymbol {x}}}^{+}(k)$ and covariance ***P*** ^+^(*k*) at each measurement
  11 $$ \begin{array}{rl} {\hat{\boldsymbol{x}}}^{+}(k) =& {\hat{\boldsymbol{x}}}^{-}(k) + {\boldsymbol{K}}(k) \left[{\boldsymbol{y}}(k) - {\boldsymbol{\Psi}}_{k} {\hat{\boldsymbol{x}}}^{-}\right], \\ {\boldsymbol{P}}^{+}(k) =& \left[{\boldsymbol{I}}-{\boldsymbol{K}}(k){\boldsymbol{\Psi}}_{k}\right]{\boldsymbol{P}}^{-}(k), \end{array}  $$
  where the superscript (+) denotes the posteriori value.
- *Propagation*: propagate both the state estimate ${\hat {\boldsymbol {x}}}(k)$ and covariance ***P***(*k*) using the posteriori estimate ${\hat {\boldsymbol {x}}}^{+}(k)$ and posteriori covariance ***P*** ^+^(*k*)
  12 $$ \begin{array}{rl} {\hat{\boldsymbol{x}}}^{-}(k+1) =& {\boldsymbol\Phi}_{k} {\hat{\boldsymbol{x}}}^{+}(k) + {\boldsymbol \Gamma}_{k} {\boldsymbol{u}}(k), \\ {\boldsymbol{P}}^{-}(k+1) =& {\boldsymbol\Phi}_{k} {\boldsymbol{P}}^{+}(k) {\boldsymbol\Phi}_{k}^{T} +{\boldsymbol \Lambda}_{k} {\boldsymbol{Q}}(k) {\boldsymbol\Lambda}_{k}^{T}. \end{array}  $$

#### Extended Kalman filter for parameter estimation

The Kalman filter is the optimum state estimator for a linear state space model observed in Gaussian noise. However, most of the biological systems are nonlinear. This renders the Kalman filter inapplicable in such scenarios. To overcome this challenge, one possible solution is to linearize the nonlinear dynamic system before applying the Kalman filter. This process of approximating the nonlinear system with a linear one while using the Kalman filter results in the EKF. It is worth to mention that although EKF is not necessarily optimal, it was adopted as a standard method to deal with nonlinear systems. The classical extended Kalman filter’s domain of convergence depends on the region where the first-order Taylor series linearization adequately approximates the nonlinear dynamics of the system. Therefore, the initializing stage requires the initial state estimate be close enough to the true state.

The general structure of the EKF is to estimate the state vector by minimizing the system variance error. Another useful application of EKF is to estimate the unknown system parameters. Augmenting the state vector to include the unknown parameters as additional states enables an efficient system identification method for nonlinear systems. The same solution is applicable to systems with uncertain parameters but it may lead to poor performance in the estimation process. The augmented system decreases the estimation error caused by imperfect model parameters. Consider the following state space model:

13 $$ \begin{array}{rcl} {\boldsymbol{x}}(k+1) &=& \boldsymbol{f}({\boldsymbol{x}}(k);{\boldsymbol\theta)}+ {\boldsymbol{w}}(k),\\ {\boldsymbol{y}}(k) &=& \boldsymbol{h}(k)+ {\boldsymbol{v}}(k), \end{array}  $$

where *k* is a time index, ***x***∈ℜ^*n*^ represents the system state vector, ***y***∈ℜ^*m*^ stands for the observation vector, ***w***∈ℜ^*n*^ and ***v***∈ℜ^*m*^ denote the system noise and the measurement noise, respectively. ***w***∈ℜ^*n*^ and ***v***∈ℜ^*m*^ are zero-mean white Gaussian stochastic processes with covariance matrices ***Q*** and ***R***, respectively. The dynamic evolution and measurements of the system are governed by the nonlinear functions ***f***:ℜ^*n*^→ℜ^*n*^ and ***h***:ℜ^*n*^→ℜ^*m*^, respectively, with ***θ*** representing the parameters of the dynamic model. Variables *n* and *m* stand for the number of states and number of measurements, respectively.

Let ***z*** denote the augmented state vector that includes the parameters of the model as additional states. The vector ***z*** is give by:

14 $$ {\boldsymbol{z}}(k)= \left[ \begin{array}{c} {\boldsymbol{x}}(k)\\ {\boldsymbol\theta}(k) \end{array}\right].  $$

The augmented version of the state space model given in Eq. (13) takes the form:

15 $${} \begin{aligned} {{\boldsymbol{z}}}(k+1) &= \left[\begin{array}{c} {\boldsymbol{x}}(k+1)\\ {\boldsymbol\theta}(k+1) \end{array}\right] = \left[\begin{array}{c} \boldsymbol{f}({{\boldsymbol{x}}(k)}) \\ {\boldsymbol\theta}(k)\end{array}\right]+ \left[\begin{array}{c} {{\boldsymbol{w}}(k)}\\ {\boldsymbol\eta}(k) \end{array}\right] \\ &= {\boldsymbol{F}}({\boldsymbol{z}}(k))+ {\boldsymbol \zeta}(k), \\ {\boldsymbol{y}}(k) &= {\boldsymbol{x}}(k) + {\boldsymbol{v}}(k), \end{aligned}  $$

where ***ζ***(*k*) denotes the zero-mean Gaussian white-noise for the augmented dynamic defined by ***F***. Constructing the augmented model in (15) assumes that the system parameters are constant (i.e., ***θ***(*k*)=***θ***). Once the augmented state equations are constructed, the standard EKF can be implemented to estimate the states of the augmented system (i.e., ***z***), which enables the joint estimation the model states ***x*** and its parameters ***θ***. For detailed derivations of the EKF, in both discrete-time and continuous time forms, the authors recommend [34]. The following steps summarize the implementation of EKF:

- *Initialization*: at *k*=0 and for given initial states ***z***_0_=[***x***_0_, ***θ***_0_]^*T*^, the initial value of the covariance matrix is given by:
  16 $$ {\boldsymbol{P}}_{0}= \left[\begin{array}{ll} {\boldsymbol{P}}_{x_{0}x_{0}} & {\boldsymbol{P}}_{x_{0}\theta_{0}} \\ {\boldsymbol{P}}_{\theta_{0}x_{0}}& {\boldsymbol{P}}_{\theta_{0}\theta_{0}} \end{array}\right].  $$
  and
  17 $$ \begin{aligned} {\hat{\boldsymbol{z}}}^{-}(0) =& E\{{\boldsymbol{z}}(0)\}={\boldsymbol{z}_{0}}, \\ {\boldsymbol{P}}^{-}(0) =& E\left\{({\boldsymbol{z}}(0)-{\boldsymbol{z}_{0}})({\boldsymbol{z}}(0)-{\boldsymbol{z}_{0}})^{T}\right\} ={\boldsymbol{P}}_{0}. \end{aligned}  $$
  The initial covariance matrices are given by
  18 $$ \begin{array}{rcl} {\boldsymbol{P}}_{x_{0}x_{0}} &=& E\left\{({\boldsymbol{x}}(0)-{\boldsymbol{x}_{0}})({\boldsymbol{x}}(0)-{\boldsymbol{x}_{0}})^{T}\right\},\\ {\boldsymbol{P}}_{x_{0}\theta_{0}} &=& E\left\{({\boldsymbol{x}}(0)-{\boldsymbol{x}_{0}})({\boldsymbol\theta}(0)-{\boldsymbol \theta_{0}})^{T}\right\},\\ {\boldsymbol{P}}_{\theta_{0}x_{0}} &=& E\left\{({\boldsymbol\theta}(0)-{\boldsymbol\theta_{0}})({\boldsymbol{x}}(0)-{\boldsymbol{x}_{0}})^{T}\right\},\\ {\boldsymbol{P}}_{\theta_{0}\theta_{0}} &=& E\left\{({\boldsymbol\theta}(0)-{\boldsymbol\theta_{0}})({\boldsymbol{\theta}}(0)-{\boldsymbol\theta_{0}})^{T}\right\}. \end{array}  $$
  Assume that ${\boldsymbol {z}}(0) \thicksim \mathcal {N}({\boldsymbol 0},P(0))$.
- *Gain*: compute the Kalman gain matrix
  19 $$ \begin{aligned} {\boldsymbol{K}}&(k) = {\boldsymbol{P}}^{-}(k) {\boldsymbol{H}}^{T}({\hat{\boldsymbol{z}}}^{-}(k)) \\ &\left[{\boldsymbol{H}}({\hat{\boldsymbol{z}}}^{-}(k)) {\boldsymbol{P}}^{-}(k) {\boldsymbol{H}}^{T}({\hat{\boldsymbol{z}}}^{-}(k) + {\boldsymbol{R}}(k)\right]^{-1}, \end{aligned}  $$
  where ${\boldsymbol {H}}\left ({\hat {\boldsymbol {z}}}^{-}(k)\right)\equiv \frac {\partial {\boldsymbol {h}}}{\partial {\boldsymbol {z}}} |_{{\hat {\boldsymbol {z}}}^{-}(k)}$.
- *Update*: update the state estimate ${\hat {\boldsymbol {z}}}^{+}(k)$ and covariance ***P*** ^+^(*k*) at each measurement
  20 $$ \begin{aligned} {\hat{\boldsymbol{z}}}^{+}(k) =~& {\hat{\boldsymbol{z}}}^{-}(k) + \\ & {\boldsymbol{K}}(k) \left[{\boldsymbol y}(k) - {\boldsymbol{h}}({\hat{\boldsymbol{z}}}^{-}(k))\right], \\ {\boldsymbol{P}}^{+}(k) =~& \left[{\boldsymbol{I}}-{\boldsymbol{K}}(k){\boldsymbol{H}}({\hat{\boldsymbol{z}}}^{-}(k))\right]{\boldsymbol P}^{-}(k). \end{aligned}  $$
- *Propagation*: propagate both the state estimate ${\hat {\boldsymbol {z}}}(k)$ and covariance ***P***(*k*) using the posteriori estimate ${\hat {\boldsymbol {z}}}^{+}(k)$ and posteriori covariance ***P*** ^+^(*k*)
  21 $$ \begin{aligned} {\hat{\boldsymbol{z}}}^{-}(k+1)&= {\boldsymbol{F}}\left({\hat{\boldsymbol{z}}}^{+}(k)\right), \\ {\boldsymbol{P}}^{-}(k+1) &= {\boldsymbol{\Omega}}\left({\hat{\boldsymbol{z}}}^{+}(k)\right) {\boldsymbol{P}}^{+}(k) {\boldsymbol{\Omega}}^{T}\left({\hat{\boldsymbol{z}}}^{+}(k)\right) \\ &\quad+ {\boldsymbol{Q}}(k), \end{aligned}  $$
  where ${\boldsymbol {\Omega }}\left ({\hat {\boldsymbol {z}}}^{+}(k)\right)\equiv \frac {\partial {\boldsymbol {F}}({\boldsymbol z})}{\partial {\boldsymbol {z}}} |_{{\hat {\boldsymbol {z}}}^{+}(k)}$.

For our model of MIN given in Eq. (1), the system dynamics (i.e., ***f***) is depicted by the gLV model defined in Eq. (4) and the observation model ***h*** is given by the identity function (i.e., ***h***(***z***(*k*))=***x***(*k*)). The system parameters vector ***θ*** captures the intrinsic growth rates and all the pairwise interaction coefficients between the *n* bacteria included in the gLV model. In particular, ***θ*** is given by:

22 $$ {\boldsymbol\theta} = \left[ g_{1}, g_{2}, \hdots g_{n}, c_{11}, c_{12}, \hdots,c_{nn} \right]^{T}.  $$

## Results and discussion

In this section, we compared SgLV-EKF with the current state-of-the-art algorithms proposed for inferring the microbial interaction network using the gLV model. In particular, EKF is compared with two similarity-based algorithms, one algorithm from the integral-based family, and two regression-based algorithms. The first similarity-based algorithm utilizes the Pearson correlation coefficient (PCC) [18], whereas the second algorithm employs the local similarity analysis [22] to quantify the similarity between time series data. For the integral-based algorithm, the gradient free Nelder-Mead algorithm [35] is used to span the parameter space for the optimal solution. For the regression-based techniques, the first regression-based algorithm was developed by Stein et al. in [11] and it employs the regularized linear regression to infer the MIN. We refer to this algorithm as the Stein’s algorithm. The second regression-based algorithm is called the learning interactions from microbial time series (LIMITS) algorithm. This algorithm was proposed in [24] and it is based on the sparse linear regression model. It is important to mention that Stein’s algorithm involves Tikhonov regularization parameters. These parameters were set to the same values used in [11]. All the experiments were performed on a Windows 8.1 system with a 3.4 GHz Intel Core i7 processor on a Matlab 8.3.0.

### Synthetic data

The MIN inference algorithms are evaluated in their ability to predict: (a) MIN; (b) Variation of the bacterial abundance levels over the time (i.e., states of the dynamic model). An important metric of any interaction network is its ability to recover the topology/structure of the simulated interaction network. Specifically, the accuracy, sensitivity, and specificity of the MIN inference algorithms in predicting the presence and/or absence of interactions. Moreover, to evaluate the ability of the MIN inference algorithms in predicting the dynamic of the bacterial system, we use the relative mean square error (MSE) as a fidelity criterion to measure the error between the observed data and the estimated bacterial abundances. In our evaluation, we define the true positive (TP) as the number of edges that are truly detected, and the false negative (FN) as the number of edges that are not detected. Similarly, if no edges are present, the number of times the algorithm mistakenly predicts the presence of an edge is defined as the false positive (FP). Otherwise, the number of times that the algorithm truly predicts the absence of an edge is defined as the true negative (TN). Sensitivity and specificity are defined as TP/(TP + FN) and TN/(TN + FP), respectively. And accuracy is defined as (TP + TN)/(TP + FN + TN + FP). Ideally, the values of sensitivity, specificity and accuracy are one. An algorithm with low sensitivity value indicates that this algorithm fails in predicting the existing edges (i.e., interactions) in the network. On the other hand, an algorithm with low specificity performance implies that the algorithm suggests the presence of edges that don’t exist in reality. We assume the absence of interaction if the absolute value of the interaction strength is less than one tenth the average of the absolute values of the nonzero elements in the simulated network (i.e., |*c*_*ij*_|<0.1).

**Fig. 1:**
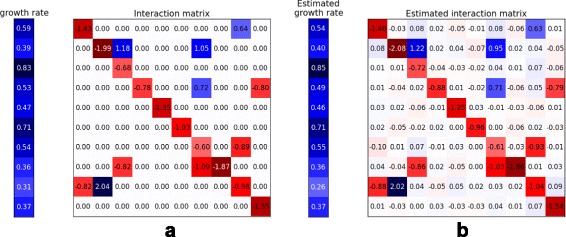
Simulation of microbial regulatory network. **a** The true values of the model parameters. **b** The inferred parameters

In order to evaluate the performance of our proposed scheme, a microbial community consisting of 10 bacteria is simulated. A number of 30 time series points (i.e., the microbial abundance levels) are generated using the stochastic gLV model (Eq. 1) with the parameters shown in Fig. 1 a. In our simulations, we perform 100 Monte-Carlo simulations, and we present the average of these experiments.

It is pertinent to mention that comparisons with similarity-based methods are limited to the evaluation of the efficiency of the algorithms to identify the presence and/or absence of interactions in the simulated networks for various dynamic/measurement noise levels. This is because similarity methods don’t include a mathematical modeling of the microbial community. Hence, similarity methods don’t enable predicting the temporal bacterial abundance profiles.

Figure 1 b shows the inferred values using EKF for the growth rate and the interaction network of the simulated microbial system. The small differences between the true values of system parameters and the inferred parameters using EKF point out that the proposed EKF-based approach is accurate in terms of estimating the true system parameters.

The performance metrics mentioned above are evaluated under the following simulation set-ups: (a) Measurement noise level (i.e., $\sigma ^{2}_{v}$); (b) System noise level (i.e., $\sigma ^{2}_{w}$).

#### Varying the measurement noise level $\sigma ^{2}_{v}$

**Fig. 2:**
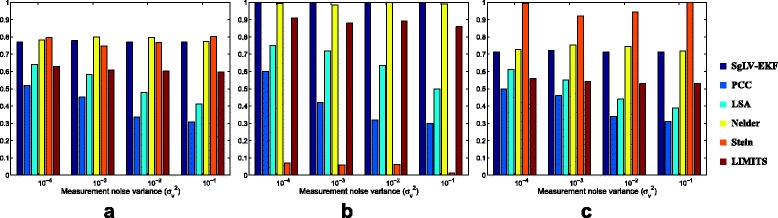
Evaluation measurements of the efficiency of the six algorithms to identify the presence and absence of interactions in the simulated networks for various measurement noise levels in terms of **a** accuracy, **b** sensitivity and **c** specificity

First, the six algorithms are tested when varying the Gaussian noise levels in the observed data with variance ranging from 10^−4^ to 10^−1^. The performance of the six algorithms in terms of their abilities to identify the simulated network is depicted in Fig. 2. As it is clearly depicted by this figure, increasing the noise variance has a slight effect on the performance of the SgLV-EKF, the Nelder and the two regression-based algorithms. On the other hand, the performance of the two similarity-based algorithms (i.e., PCC and LSA) degrades significantly by increasing the noise power. Moreover, similarity-based algorithms show the least accurate performance compared to the other algorithms. The performance of similarity-based techniques may be attributed to two main reasons. The first reason is that the abundance profiles of two microorganisms may be correlated even they don’t interact directly. For example, if bacteria A and B do not assume a direct interaction, but both of them rely on the products of bacteria C, then the abundance profiles of A and B are expected to be correlated. The second reason is that the bacterial abundance data provided by the sequencing-based techniques represent the relative fraction of the bacterial abundances rather than their absolute abundances. This compositional nature of the bacterial profiles can lead to unreliable results [36].

For the regression-based algorithms, Stein’s algorithm fails to detect the majority of the interactions as depicted from the very low sensitivity values in Fig. 2 b, whereas LIMITS algorithm provides more reliable results with consistence accuracy performance around 60%. However, SgLV-EKF outperforms both Stein’s and LIMITS algorithms. SgLV-EKF and Nelder’s algorithm yield close and stable results over different variance values. However, the execution time of Nelder algorithm is approximately 80 times higher than the SgLV-EKF execution time as it is pointed out by Table 1.

**Table 1:** Average execution time for various methods (seconds)

	SgLV-EKF	Nelder	Stein	LIMITS
Execution Time	2.11	161.62	0.06	1.4

**Fig. 3:**
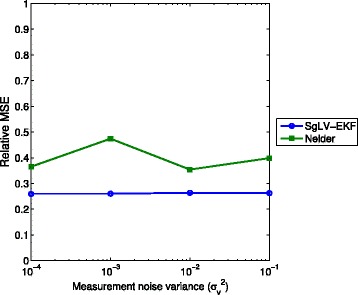
Relative MSE performance under different measurement noise levels

The relative MSE of the predicted bacterial abundance levels is depicted in Fig. 3. The noise level has a negligible effect on the relative MSE of SgLV-EKF and Nelder’s algorithm. Both SgLV-EKF and Nelder’s algorithm exhibit low MSE errors. The estimated parameters resulted from both Stein’s and LIMITS methods lie in the unstable region of the dynamic system. Therefore, they present an infinite MSE error.

#### Varying the dynamic noise level $\sigma ^{2}_{w}$

This section evaluates the algorithms performance against uncertainties in the dynamic model. This uncertainty is modeled by a zero mean white Gaussian noise with variance varying from 10^−7^ to 10^−1^.

**Fig. 4:**
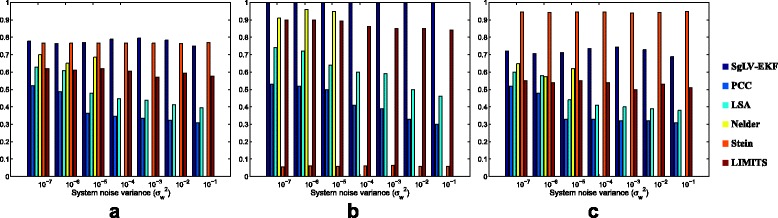
Evaluation measurements of the efficiency of the six algorithms to identify the presence and absence of interactions in the simulated networks for various dynamic noise levels in terms of **a** accuracy, **b** sensitivity and **c** specificity

Figure 4 presents the accuracy, sensitivity, and specificity of the six MIN inference algorithms. Since our scheme takes into account the randomness in the dynamic model, SgLV-EKF outperforms the other five algorithms in identifying the structure of the interaction network. Moreover, SgLV-EKF provides a robust and reliable performance against the uncertainty in the dynamic model and it exhibits an average accuracy higher than than 75%. On the other hand, due to the presence of a small amount of noise in the dynamic model, the estimation using the other five algorithms is unreliable and inconsistent. In particular, the two similarity-based algorithms show a significant reduction in their accuracy performance due to the increase in the process noise power. For example, for noise level exceeding 10^−4^, PCC and LSA achieve an average accuracy of only 45% and 35%, respectively. Similar to the results in the previous section, Stein’s method failed in inferring the existing interactions as illustrated by the very low sensitivity values in Fig. 4 b. For noise power values larger than 10^−4^, Nelder’s method diverged and failed in providing any estimate of the model’s parameters. The divergence of Nelder’s algorithm combined with the robust performance of the SgLV-EKF algorithm justify our approach of replacing the conventional ODE-based gLV model with a stochastic gLV model that accounts for uncertainties in the system model.

**Fig. 5:**
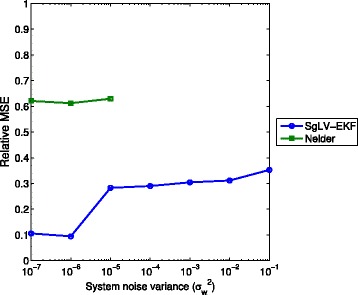
Relative MSE performance under different dynamic noise levels. For $\sigma ^{2}_{w}$ is larger than 10^−4^, Nelder’s algorithm diverges

Figure 5 presents the relative MSE for the SgLV-EKF and Nelder’s algorithms. The results of Stein’s and LIMITS algorithms are omitted here for the same reason mentioned in the previous section. SgLV-EKF shows a consistent performance against system noise.

### Real data

To further demonstrate the capability of SgLV-EKF algorithm in inferring the microbial interaction networks, we considered two realistic time series datasets. Recently, an investigation to assess the effect of antibiotics on the intestinal microbial community infected with C. difficile was carried out in [37]. In this study, DNA sequences were taken from the cecum and the ileum of 9 mice models. The sequences generated from this study were analyzed in [11] to obtain the OTUs profiles of each sample. The OTU assignment retains the ten most abundant genera (listed in Table 2) in addition to C. difficile which together account for approximately 90% of the total 16S rRNA sequences. In this paper, the time series data belonging to two mice under different conditions were considered.

**Table 2:** OTUs that are considered in the construction of the MINs of the two realistic datasets

OUT 1:	Barnesiella
OUT 2:	Undefined genus of Lachnospiraceae
OUT 3:	Unclassified Lachnospiraceae
OUT 4:	Other
OUT 5:	Blautia
OUT 6:	Undefined genus of unclassified Mollicutes
OUT 7:	Akkermansia
OUT 8:	Coprobacillus
OUT 9:	Clostridium difficile
OUT 10:	Enterococcus
OUT 11:	Undefined genus of Enterobacteriaceae

It is pertinent to remember that, for the moment, no complete microbial interactions database reference is available to objectively evaluate the results obtained based on real data sets. However, the results can be assessed by evaluating their consistency with biological assumptions and their agreement with previous studies. For more accurate evaluation, the identified interactions need further analysis via high-throughput experiments. Also, since the constructed MINs include only a subset (i.e., 11 OTUs) of the total OTUs presented in the samples, an edge between two microbes may not necessarily indicate a direct interaction. For example, if two microbes are co-regulated by another microbe which is not included in the 11 OTUs, these two microbes may exhibit an interaction between them.

#### Dataset-1: Gut microbiota of mouse model infected by C. difficile

**Fig. 6:**
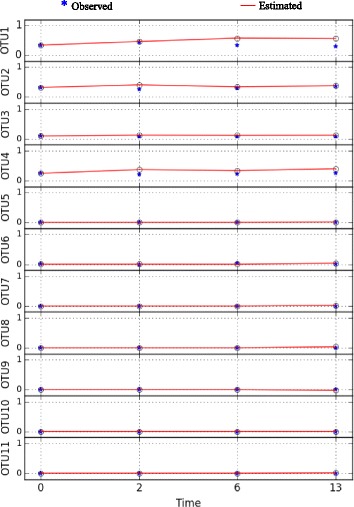
Time series of observed and predicted bacterial abundance levels in relation to Dataset-1

This dataset consists of 4 time points taken over two weeks and it belongs to the mouse with ID 8. This mouse received spores of C. difficile and was used to determine the impact of the pathogen (i.e., C. difficile) on the native gut microbiota. Figure 6 depicts the measured bacterial abundance level time series data **x**_*i*_ and its predicted values $\hat {\mathbf {x}}_{i}$. The results show that the SgLV-EKF was successful in tracking the bacterial abundance level.

**Fig. 7:**
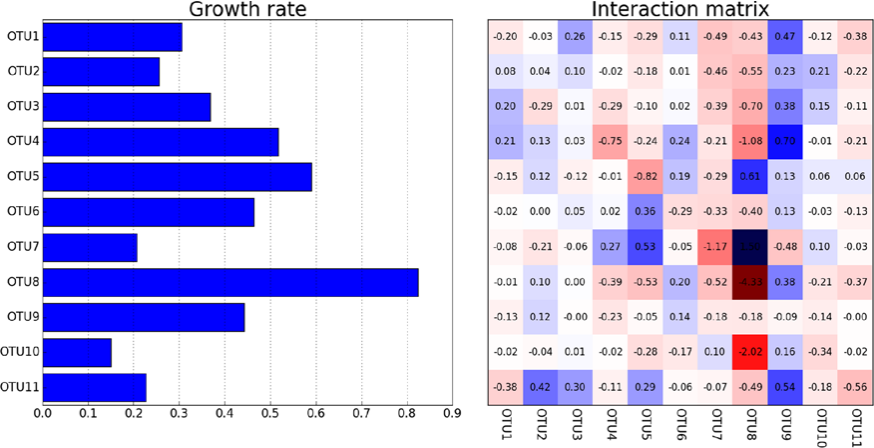
Inferred growth rates and interaction matrix in relation to Dataset-1

The predicted values for the growth rates and the MIN are depicted in Fig. 7. The inferred growth rates are consistent with the biological assumptions in the sense that they are all positive. Moreover, their range [0.2−0.83] agrees with typical growth rate ranges [0.43−1.46] [25] and [0.2−0.9] [11]. The comparable growth rates for the bacterial populations may indicate the existence of a balance state in the bacterial ecosystem. In other words, the environment is not dominated by one or few species with significantly higher growth rates.

**Fig. 8:**
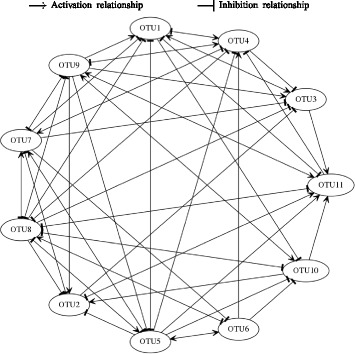
Inferred microbial interaction network in relation to Dataset-1

The negative values of the diagonal elements in the inferred interaction matrix Fig. 7 are consistent with the underlying biology. This is because the negative values indicate that each species would reach the carrying capacity even in the absence of the other species [11]. Intriguingly, even Coprobacillus exhibits low abundance levels as shown in Fig. 6, the inferred MIN suggests Coprobacillus as the bacteria with the strongest interactions (i.e., larger interaction coefficients values) with other members in the microbial community. In particular, Coprobacillus inhibits all other microbes except Akkermansia and Blautia. Interstingly, all the bacteria exhibit inhibitory activity against C. difficile except Enterococcus, Undefined genus of Lachnospiraceae, and Undefined genus of unclassified Mollicutes which positively interact with the pathogen. This positive interaction agrees with previous results in [38]. The predicted MIN suggests C. difficile to negatively impact Blautia and Coprobacillus. This complies with the findings in [39] that show that both Blautia and Coprobacillus are among the top genera that are depleted in patients infected by C. difficile. Moreover, the inferred MIN shows that Barnesiella is negativelly interacting with Enterococcus. This agrees with the results found in [40]. The constructed microbial interaction network is displayed in Fig. 8.

#### Dataset 2: Gut microbiota of mouse model infected by C. difficile and treated with clindamycin

**Fig. 9:**
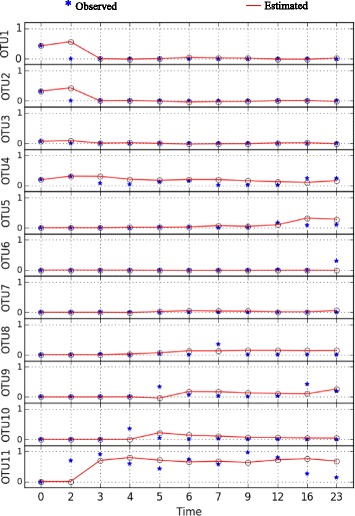
Time series of observed and predicted bacterial abundance levels in relation to Dataset-2

This dataset consists of 11 time points taken over 23 days and it belongs to the mouse with ID 9. At the first day of experiment, this mouse was injected with a single dose of clindamycin, and on the following day received spores of C. difficile. This experiment aimed to investigate the impact of the antibiotic (i.e., clindamycin) on the intestinal bacterial structure. The bacterial abundance levels and their estimated values are presented in Fig. 9. It is clear that the inferred model provides a fairly good prediction of the bacterial abundance data. By comparing the abundance levels in the two datasets, it is clear that the clindamycin antibiotic alters the structure of the microbial community. In particular, Barnesiella and the undefined genus of Lachnospiraceae are severely depleted in response to clindamycin. On the other hand, Enterococcus, the undefined genus of Enterobacteriaceae and more importantly C. difficile exhibit an increase in their abundance levels. This suggests that the induced dysbiosis in the bacterial community from its normal state due to the clindamycin antibiotic facilitates the colonization of C. difficile.

**Fig. 10:**
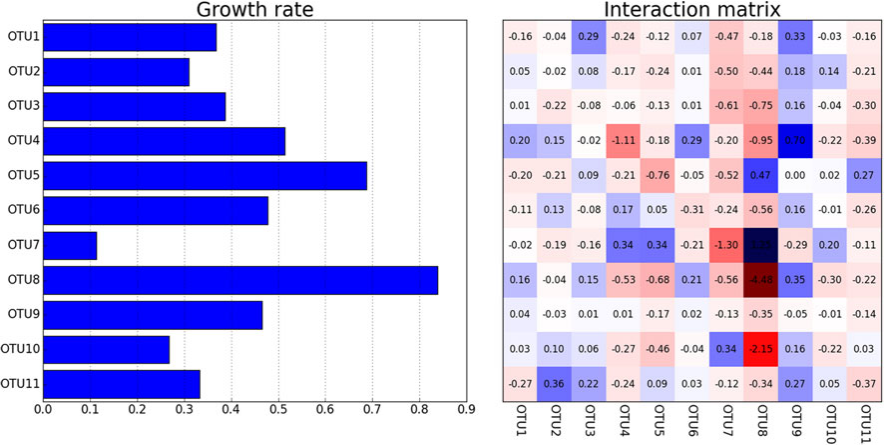
Inferred growth rates and interaction matrix in relation to Dataset-2

**Fig. 11:**
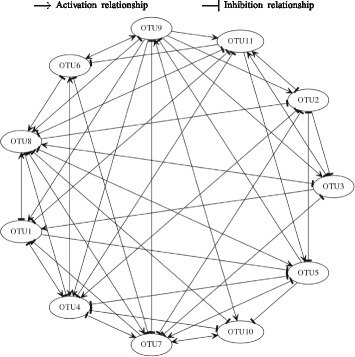
Inferred microbial interaction network in relation to Dataset-2

The inferred interaction matrix shown in Fig. 10 supports these findings. For example, the simultaneous increase in the abundance levels of Enterococcus, the undefined genus of Enterobacteriaceae and C. difficile can be explained by the mutualistic (i.e., +|+) relationships between them. The inferred growth rates shown in Fig. 10 are all positive and ranging between 0.2 and 0.89. This complies with the biological assumption as discussed earlier. The obtained microbial interaction network is shown in Fig. 11.

## Conclusions

In this work, we propose the SgLV-EKF algorithm to model the microbial dynamic and infer their interactions. In particular, we replace the conventional model of MIN formulated as a gLV dynamic model with a with a stochastic gLV model. The introduced stochastic model accounts for the uncertainties in the model and/or measurements. The proposed stochastic model accounts for the uncertainty in the model by adding a noise term in the dynamic equation. Moreover, to deal with the challenges of inferring MIN (i.e., nonlinear dynamics and limited number of observations), we propose EKF to jointly estimate the bacterial abundance levels and their interactions. The online and recursive nature of EKF enables fast and reliable estimation of the model’s parameters from short time series data.

The performance of the proposed SgLV-EKF algorithm is compared with two similarity-based algorithms (i.e., PCC and LSA), one integral-based algorithm (i.e., Nelder’s algorithm) and two regression-based algorithms (i.e., Stein’s and LIMITS algorithms) in the presence of synthetic as well as realistic data sets by varying the noise levels in both the measurements and dynamic model.

It is observed that Stein’s algorithm, an example of regression-based algorithms, is computationally efficient. However, it consistently exhibits a very low sensitivity indicating its failure to detect the majority of the interactions. This renders Stein’s algorithm unreliable and inaccurate for estimating the MIN. This inaccuracy is because its sensitivity to the selection of the regularization parameters and the approximation of the derivatives in the ODE model. Particularly, the authors in [11] applied the forward difference to estimate the derivatives, which represents a coarse approximation of the slope of the bacterial abundance profiles. The LIMITS algorithm, a second example of regression-based algorithms, achieves more reliable and consistent performance compared to Stein’s algorithm. However, this improvement comes at the cost of increased computational time. The reason behind increasing the execution time of LIMITS algorithm is the bagging procedure implemented in LIMITS to reduce the bias caused by the ‘errors-in-variables’ problem [41]. Similar to Stein’s algorithm, the performance of LIMITS algorithm is sensitive to the accuracy of the approximation used to evaluate the derivatives in the ODE model.

Nelder’s algorithm, an implementation of the integral-based approaches, offers close results to the SgLV-EKF when varying the measurements noise. However, Nelder’s algorithm failed to compensate for randomness in the dynamic system as the algorithm diverges in the presence of noise with power exceeding 10^−4^. Moreover, SgLV-EKF is more computationally efficient due to its sequential structure. The main virtue of similarity-based algorithms is that they are computationally efficient. However, similarity-based methods can capture only pairwise relationships between species. This renders these methods incapable of handling the existing complex interactions in microbial communities.

Overall, the robustness against uncertainty in measurements and/or model, the enhanced accuracy relative to the state-of-the-art algorithms, and the reduced computational time make SgLV-EKF a promising approach to model the microbial dynamics and infer the interactions among microbes.

## Abbreviations

EKF: Extended Kalman Filter
gLV: Generalized Lotka-Volterra
LIMITS: Learning interactions from MIcrobial time series
LSA: Local similarity analysis
MIN: Microbial interaction network
ODE: Ordinary differential equation OTU: Operational taxonomic unit
PCC: Pearson correlation coefficient
SgLV-EKF: Stochastic gLV model with extended Kalman filter (EKF)

## References

[1] Fujimura KE, Slusher NA, Cabana MD, Lynch SV (2010). Role of the gut microbiota in defining human health. Expert Rev Anti-Infect Ther.

[2] Flint HJ (2011). Obesity and the gut microbiota. J Clin Gastroenterol.

[3] Turnbaugh PJ, Hamady M, Yatsunenko T, Cantarel BL, Duncan A, Ley RE (2009). A core gut microbiome in obese and lean twins. Nature.

[4] Ridaura VK, Faith JJ, Rey FE, Cheng J, Duncan AE, Kau AL (2013). Gut microbiota from twins discordant for obesity modulate metabolism in mice. Science.

[5] Larsen N, Vogensen FK, Van Den Berg F, Nielsen DS, Andreasen AS, Pedersen BK (2010). Gut microbiota in human adults with type 2 diabetes differs from non-diabetic adults. PloS ONE.

[6] Morgan XC, Tickle TL, Sokol H, Gevers D, Devaney KL, Ward DV (2012). Dysfunction of the intestinal microbiome in inflammatory bowel disease and treatment. Genome Biol.

[7] Moore W, Moore LH (1995). Intestinal floras of populations that have a high risk of colon cancer. Appl Environ Microbiol.

[8] Ahn J, Sinha R, Pei Z, Dominianni C, Wu J, Shi J, et al. Human gut microbiome and risk of colorectal cancer. J Natl Cancer Inst. 2013. doi:10.1093/jnci/djt300. 10.1093/jnci/djt300 PMC3866154 24316595

[9] Faust K, Raes J (2012). Microbial interactions: from networks to models. Nat Rev Microbiol.

[10] Dethlefsen L, Huse S, Sogin ML, Relman DA (2008). The pervasive effects of an antibiotic on the human gut microbiota, as revealed by deep 16S rRNA sequencing. PLoS Biology.

[11] Stein RR, Bucci V, Toussaint NC, Buffie CG, Rätsch G, Pamer EG (2013). Ecological modeling from time-series inference: insight into dynamics and stability of intestinal microbiota. PLoS Comput Biol.

[12] Faust K, Raes J (2012). Microbial interactions: from networks to models. Nat Rev Microbiol.

[13] Bucci V, Nadell CD, Xavier JB (2011). The evolution of bacteriocin production in bacterial biofilms. Am Nat.

[14] Klitgord N, Segre D (2010). Environments that induce synthetic microbial ecosystems. PLoS Comput Biol.

[15] Khosravi A, Mazmanian SK (2013). Disruption of the gut microbiome as a risk factor for microbial infections. Curr Opin Microbiol.

[16] Faust K, Sathirapongsasuti JF, Izard J, Segata N, Gevers D, Raes J (2012). Microbial co-occurrence relationships in the human microbiome. PLoS Comput Biol.

[17] Song HS, Cannon WR, Beliaev AS, Konopka A (2014). Mathematical modeling of microbial community dynamics: a methodological review. Processes.

[18] David LA, Materna AC, Friedman J, Campos-Baptista MI, Blackburn MC, Perrotta A (2014). Host lifestyle affects human microbiota on daily timescales. Genome Biol.

[19] Eiler A, Heinrich F, Bertilsson S (2012). Coherent dynamics and association networks among lake bacterioplankton taxa. ISME J.

[20] Fuhrman JA, Steele JA (2008). Community structure of marine bacterioplankton: patterns, networks, and relationships to function. Aquat Microb Ecol.

[21] Ruan Q, Dutta D, Schwalbach MS, Steele JA, Fuhrman JA, Sun F (2006). Local similarity analysis reveals unique associations among marine bacterioplankton species and environmental factors. Bioinformatics.

[22] Xia LC, Steele JA, Cram JA, Cardon ZG, Simmons SL, Vallino JJ (2011). Extended local similarity analysis (eLSA) of microbial community and other time series data with replicates. BMC Syst Biol.

[23] Mounier J, Monnet C, Vallaeys T, Arditi R, Sarthou AS, Hélias A (2008). Microbial interactions within a cheese microbial community. Appl Environ Microbiol.

[24] Fisher CK, Mehta P (2014). Identifying Keystone Species in the Human Gut Microbiome from Metagenomic Timeseries Using Sparse Linear Regression. PLoS ONE.

[25] Marino S, Baxter NT, Huffnagle GB, Petrosino JF, Schloss PD (2014). Mathematical modeling of primary succession of murine intestinal microbiota. Proc Natl Acad Sci.

[26] Tsai KY, Wang FS (2005). Evolutionary optimization with data collocation for reverse engineering of biological networks. Bioinformatics.

[27] Voit E, Chou IC (2010). Parameter estimation in canonical biological systems models. Int J Syst Synthetic Biol.

[28] Chou IC, Martens H, Voit EO (2006). Parameter estimation in biochemical systems models with alternating regression. Theor Biol Med Model.

[29] Zhan C, Yeung LF (2011). Parameter estimation in systems biology models using spline approximation. BMC Syst Biol.

[30] Corigliano A, Mariani S (2004). Parameter identification in explicit structural dynamics: performance of the extended Kalman filter. Comput Methods Appl Mech Eng.

[31] Lillacci G, Khammash M (2010). Parameter estimation and model selection in computational biology. PLoS Comput Biol.

[32] Wang Z, Liu X, Liu Y, Liang J, Vinciotti V (2009). An extended Kalman filtering approach to modeling nonlinear dynamic gene regulatory networks via short gene expression time series. IEEE/ACM Trans Comput Biol Bioinformatics (TCBB).

[33] Albiol J, Robuste J, Casas C, Poch M (1993). Biomass estimation in plant cell cultures using an extended Kalman filter. Biotechnol Prog.

[34] Crassidis JL, Junkins JL (2011). Optimal Estimation of Dynamic Systems. Chapman & Hall/CRC Applied Mathematics & Nonlinear Science.

[35] Nelder JA, Mead R (1965). A simplex method for function minimization. ComputerJournal.

[36] Friedman J, Alm EJ (2012). Inferring correlation networks from genomic survey data. PLoS Comput Biol.

[37] Buffie CG, Jarchum I, Equinda M, Lipuma L, Gobourne A, Viale A (2012). Profound alterations of intestinal microbiota following a single dose of clindamycin results in sustained susceptibility to Clostridium difficile-induced colitis. Infect Immun.

[38] Donskey CJ, Ray AJ, Hoyen CK, Fuldauer PD, Aron DC, Salvator A (2003). Colonization and infection with multiple nosocomial pathogens among patients colonized with vancomycin-resistant enterococcus. Infect Control Hosp Epidemiol.

[39] Antharam VC, Li EC, Ishmael A, Sharma A, Mai V, Rand KH (2013). Intestinal dysbiosis and depletion of butyrogenic bacteria in Clostridium difficile infection and nosocomial diarrhea. J Clin Microbiol.

[40] Ubeda C, Bucci V, Caballero S, Djukovic A, Toussaint NC (2013). Intestinal microbiota containing Barnesiella species cures vancomycin-resistant Enterococcus faecium colonization. Infect Immun.

[41] Fuller WA (1980). Properties of some estimators for the errors-in-variables model. Ann Stat.

